# Water Translocation and Photosynthetic Responses in Clones of Kentucky Bluegrass to Heterogeneous Water Supply

**DOI:** 10.3390/plants14050826

**Published:** 2025-03-06

**Authors:** Jia Jiang, Chen Wang, Along Chen, Fuchun Xie, Yajun Chen

**Affiliations:** 1 College of Animal Science and Technology, Northeast Agricultural University, Harbin 150030, China; a1416351354@163.com (J.J.); s230501001@neau.edu.cn (C.W.); 2 College of Horticulture, Northeast Agricultural University, Harbin 150030, China; s230401065@neau.edu.cn

**Keywords:** clonal ramets, stable isotope, PEG6000

## Abstract

Drought stress is the most common threat to plant growth, while physiological integration can significantly enhance the drought tolerance of clonal plants, making it essential to research the behavior of clones under drought conditions and explore the potential applications of clonal plants. This study applied polyethylene-glycol-6000-induced stress to proximal, middle and distal clonal ramets of Kentucky bluegrass (*Poa pratensis* L.) and used an isotope labeling technique to evaluate the water physiological integration and photosynthetic capacity. When the proximal ramet was subjected to drought stress treatment, the decrease in ^2^H isotopes in the roots from 4 h to 6 h was significantly smaller than the increase in ^2^H isotopes in their own leaves. Additionally, the reductions in δ^2^H values of middle and distal ramets roots were 4.14 and 2.6 times greater, respectively, than the increases in their respective leaf δ^2^H values. The results indicate that under drought stress, water physiological integration was observed among different clonal ramets. In addition, drought stress inhibits the photosynthetic-related indicators in clonal ramets, with varying degrees of response and trends in photosynthetic characteristics among different clonal ramets. The proximal ramet treatment group, treated with polyethylene glycol 6000, was most affected by drought stress, while the distal ramet treatment group was least affected. The proximal ramet treatment group, treated with polyethylene glycol 6000, showed a decrease in water use efficiency after 6 h of drought treatment, while the other groups exhibited some increase. This indicates differences in water utilization and regulation among the different clonal ramets under drought stress. This study holds significant theoretical importance for exploring the characteristics of physiological integration and the photosynthetic mechanisms of Kentucky bluegrass clones under drought stress.

## 1. Introduction

Numerous changes in morphological, metabolic and physiological traits are induced in plants by drought stress [1]. Drought stress can negatively affect the physiology of plants, causing a series of problems, including leaf yellowing, cell membrane injury and photosynthetic rate reduction [2,3]. Water is a key factor in plant growth, affecting physiological activities such as photosynthesis and nutrient absorption. To adapt to water-deficient environments, plants have evolved a variety of strategies, such as activating the antioxidant defense system, inducing osmotic adjustment (OA), and promoting stomatal closure [4,5,6,7]. In addition, clonal plants can improve their growth, development and survival rate under drought conditions through effective integration and utilization of limited resources [8].

Clonal plants are those that reproduce asexually via propagules (such as shoots and buds) connected to the parent, resulting in new individuals that are nearly identical to the parental genotype [9]. Physiological integration, also referred to as clonal integration, is a unique survival strategy utilized by clonal plants when faced with difficult environmental conditions. These ramets are often linked by rhizomes, stolons, or roots, allowing for the transfer and sharing of resources such as water and nutrients [8]. Physiological integration between parent and offspring in clonal plants facilitates the establishment of new individuals [10]. Additionally, under certain circumstances, mature clones may exhibit water physiological integration. During drought conditions, water can be transferred from the parent plant to its water-stressed clones, thereby boosting the overall drought tolerance of the plant and achieving water integration [10]. Previous studies have shown that cutting the connecting stolon between *Zoysia japonica* clones negatively affects their growth and morphology, leading to incomplete physiological integration of carbon and water resources among the clones [11]. Water physiological integration plays a vital role in enabling clonal plants to endure drought stress, making it essential to thoroughly understand the underlying mechanisms.

Water management and utilization in clonal plants can significantly benefit their growth in drought conditions. The water status of plants during periods of water stress significantly impacts photosynthetic processes [12,13]. However, there remains a limited number of studies examining the effects of water stress on photosynthesis, specifically in clonal plants.

In addition to enhancing aesthetics and providing spaces for leisure, recreation, and sports, lawns play a crucial role in air purification, water conservation, and soil preservation [14]. Turfgrass, characterized by a lengthy growth period, extensive leaf area, rapid growth rate, and a robust root system, requires significant amounts of water; thus, water availability strongly influences its growth, development, and overall health [15]. Kentucky bluegrass (*Poa pratensis* L.) is a well-known cool-season turfgrass recognized globally [15]. Its rhizomes possess a strong ability to penetrate the soil, allowing the plant to effectively absorb water and nutrients, even in dry and compacted conditions [16]. During extended drought periods, Kentucky bluegrass tends to extend its roots and stems deeper into the soil to access more abundant water resources. This behavior illustrates the plant’s ability to adapt its water physiology through phenotypic plasticity, which supports the growth of clonal ramets and enables it to thrive in optimal ecological niches [17]. This study aims to explore how water regulation in Kentucky bluegrass clonal ramets during drought conditions influences photosynthetic activities.

## 2. Results

### 2.1. Water Translocation Characteristics in Clonal Ramets When Proximal Ramet Exposed to Drought Stress

**Figure 1:**
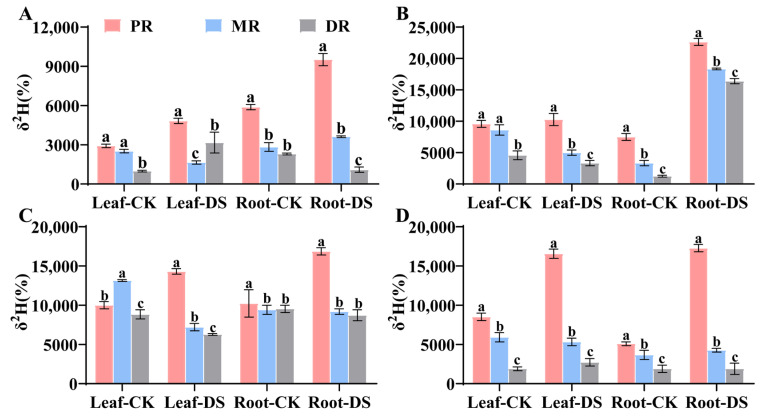
The pattern of ^2^H_2_O transfer between clones by the PR’s root under drought stress. (**A**–**D**): treatment of PR for 2 h, 4 h, 6 h and 8 h. Data in the figures indicate mean of four replicates (±SD), and different letters on error bars indicate significant difference at *p* < 0.05.

The proximal ramet (PR) of Kentucky bluegrass was subjected to a drought stress treatment at different time gradients, resulting in significant changes in the δ^2^H values of the roots and leaves of each ramets (Figure 1). At 2 h of the drought treatment, the δ^2^H value of the PR, as the point of ^2^H isotope introduction, was the highest, and the difference compared to the roots of other ramets was significant (*p* < 0.05). As the duration of the drought stress increased, the ^2^H abundance in the roots of the MR and DR exhibited a trend of first increasing and then decreasing. Additionally, the decrease in ^2^H isotopes in the roots from 4 h to 6 h was significantly smaller than the increase in ^2^H isotopes in their own leaves, with the reductions in δ^2^H values of MR and DR roots being 4.14 and 2.6 times greater than the increases in their respective leaf δ^2^H values. After 8 h of cultivation, the δ^2^H values of the PR roots were significantly higher than those of the roots of other ramets (Figure 1D). Meanwhile, the δ^2^H values within the ramets were transmitted from the roots to the leaves, and the δ^2^H values of the leaves of the PR and DR ramets in the drought treatment group showed varying degrees of increase over time (Figure 1). Among these, the δ^2^H values in the leaves of PR reached their maximum at 8 h, which was significantly different from those of MR and DR (*p* < 0.05). However, the δ^2^H value of the MR leaves exhibited a slight decrease from 6 h to 8 h as time progressed.

### 2.2. Water Translocation Characteristics in Clonal Ramets When Middle Ramet Exposed to Drought Stress

**Figure 2:**
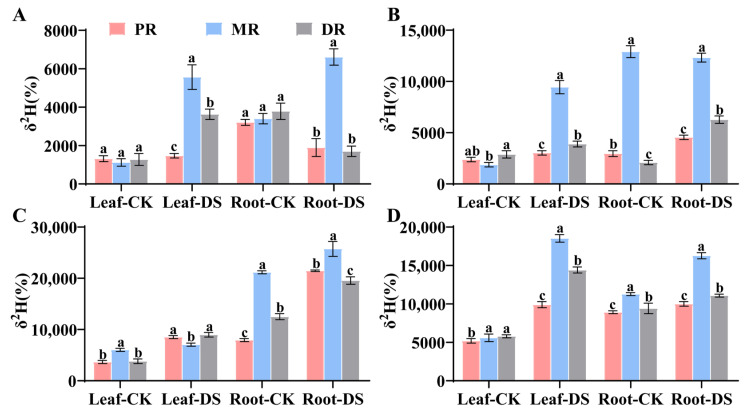
The pattern of ^2^H_2_O transfer between clones by the MR’s root under drought stress. (**A**–**D**): treatment of MR for 2 h, 4 h, 6 h and 8 h. Data in the figures indicate mean of four replicates (±SD), and different letters on error bars indicate significant difference at *p* < 0.05.

The middle ramet (MR) of Kentucky bluegrass was subjected to the drought stress treatment at different time gradients, resulting in distinct trends in the δ^2^H values of the roots and leaves of PR, MR, and DR (Figure 2). Under drought stress conditions, as the duration of stress increased, the δ^2^H value of MR leaves showed a trend of first increasing, then decreasing, and finally increasing again, reaching its maximum at 8 h. In contrast, the δ^2^H value of the MR roots peaked at 6 h and decreased from 6 h to 8 h. As the drought stress duration extended, the δ^2^H value of DR leaves exhibited an increasing trend; however, there was little change from 2 h to 4 h, followed by a sharp increase from 6 h to 8 h (Figure 2). The δ^2^H value of DR roots displayed a trend of first rising and then falling, with the highest abundance at 6 h. The trend in the δ^2^H value of PR roots was similar to that of the DR roots.

### 2.3. Water Translocation Characteristics in Clonal Ramets When Distal Ramet Exposed to Drought Stress

**Figure 3:**
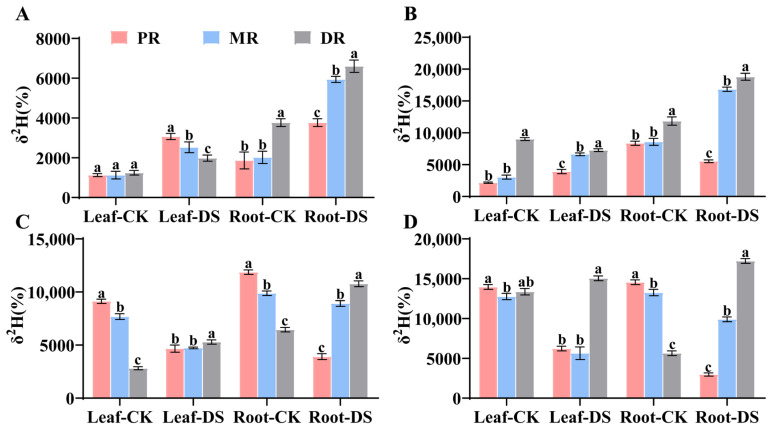
The pattern of ^2^H_2_O transfer between clones by the DR’s root under drought stress. (**A**–**D**): treatment of DR for 2 h, 4 h, 6 h and 8 h. Data in the figures indicate mean of four replicates (±SD), and different letters on error bars indicate significant difference at *p* < 0.05.

The distal ramet (DR) of Kentucky bluegrass was subjected to the drought stress treatment at different time gradients, resulting in significant changes in the δ^2^H values of the roots and leaves over time (Figure 3). After 2 h of cultivation, the δ^2^H value of the DR roots was the highest in both the treatment and control groups and was significantly different from the roots of other ramets (*p* < 0.05). Furthermore, the δ^2^H values of the DR roots in the drought stress treatment group were higher than those in the control group. Under drought stress conditions, the δ^2^H value of the DR roots showed a trend of first increasing and then decreasing, reaching its maximum at 6 h. The δ^2^H values of both the DR roots and leaves decreased from 4 h to 6 h of drought stress. Compared with 4 h, the δ^2^H values of both the DR roots and leaves showed reductions of 0.427 and 0.274 times under 6 h drought stress, respectively; the decrease in the δ^2^H value of the DR leaves was significantly lower than that of the roots. In the treatment group, the δ^2^H values of PR and MR roots showed a trend of first increasing and then decreasing as the cultivation time extended. Compared to the 2 h drought stress, the δ^2^H value of the MR roots increased by 0.67 times after 8 h of drought treatment, while the δ^2^H value of the PR roots decreased by 0.21 times.

### 2.4. Changes in Photosynthetic Characteristics and Water Use Efficiency in Clonal Ramets Under Drought Stress

**Figure 4:**
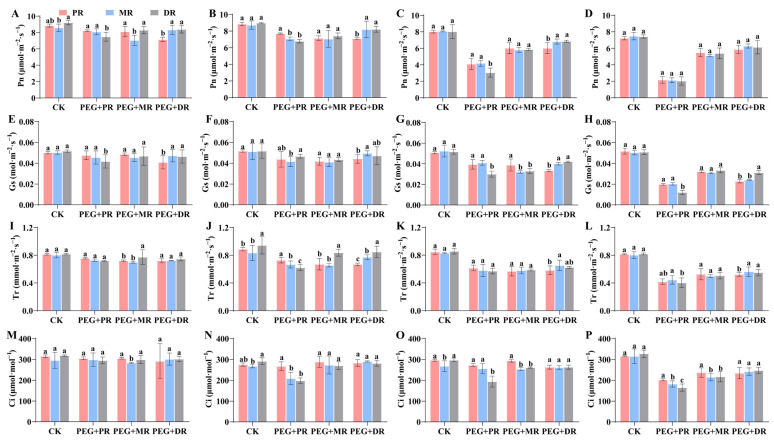
Changes in photosynthetic rate (Pn, (**A**–**D**)), stomatal conductance (Gs, (**E**–**H**)) transpiration rate (Tr, (**I**–**L**)) and intercellular CO_2_ concentration (Ci, (**M**–**P**)) of all clonal ramets under PEG6000 conditions over 2 h (**A**,**E**,**I**,**M**), 4 h (**B**,**F**,**J**,**N**), 6 h (**C**,**G**,**K**,**O**) and 8 h (**D**,**H**,**L**,**P**). CK stands for control group, PEG+PR means drought treatment of the PR of the clonal plant, PEG+MR means drought treatment of the MR of the clonal plant, PEG+DR means drought treatment of the DR of the clonal plant. Data in the figures indicate mean of four replicates (±SD), and different letters on error bars indicate significant difference at *p* < 0.05.

In the 2 h treatment (Figure 4A), the photosynthetic rate (Pn) of CK was significantly higher than that of the various drought treatment groups (PEG+PR, PEG+MR, PEG+DR). As the duration of the drought treatment increased, the Pn of each drought treatment group continued to decline, with significant differences between groups (*p* < 0.05). Notably, the rate of decline in Pn varied among different clonal ramets, with the PEG+DR group exhibiting a relatively gradual decline, while the PEG+PR group showed a marked acceleration in the decrease in Pn between 4 h and 6 h. The trend in stomatal conductance (Gs) was similar; at 2 h (Figure 4E), the Gs of the CK group was higher than that of the drought treatment groups. As the treatment time increased, the Gs of each drought treatment group gradually decreased, indicating that drought stress inhibited stomatal opening and affected gas exchange. During this process, the decline in Gs of the PEG+MR group from 6 h to 8 h was significantly smaller than that of the PEG+PR and PEG+DR groups. At 2 h (Figure 4I), the transpiration rate (Tr) of the drought treatment groups was lower than that of the CK group, and the Tr continued to decrease across different drought stress durations. Different clonal ramets also exhibited variations in Tr changes; the PEG+DR group showed a relatively uniform decline in the Tr throughout the drought treatment period, while the PEG+PR group had a slow decline initially, followed by a sharp decline. The intercellular CO_2_ concentration (Ci) at 2 h (Figure 4M) showed no significant difference between the drought treatment groups and the CK group, but as the drought treatment duration increased, the Ci of each drought treatment group gradually decreased. During this change, the Ci of the PEG+DR group exhibited the smallest reduction.

**Figure 5:**
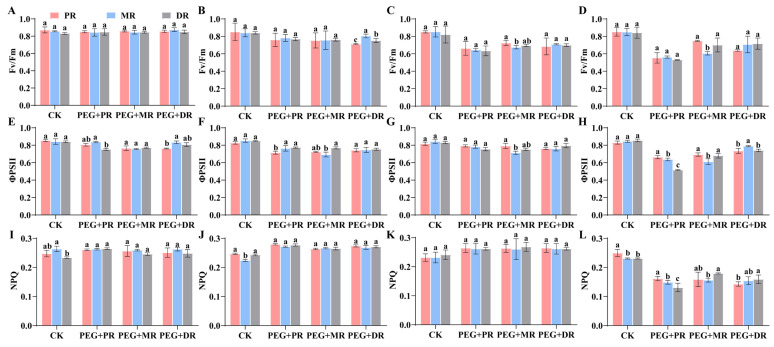
Changes in maximal photochemical efficiency of PSII (Fv/Fm, (**A**–**D**)), actual photochemical quantum efficiency (ΦPSII, (**E**–**H**)) and non-photochemical quenching (NPQ, (**I**–**L**)) of all clonal ramets under PEG6000 conditions over 2 h (**A**,**E**,**I**), 4 h (**B**,**F**,**J**), 6 h (**C**,**G**,**K**) and 8 h (**D**,**H**,**L**). CK stands for control group, PEG+PR means drought treatment of the PR of the clonal plant, PEG+MR means drought treatment of the MR of the clonal plant, and PEG+DR means drought treatment of the DR of the clonal plant. Data in the figures indicate mean of four replicates (±SD), and different letters on error bars indicate significant difference at *p* < 0.05.

In the drought treatment for 2 h (Figure 5), there was no significant difference in maximal photochemical efficiency of PSII (Fv/Fm), actual photochemical quantum efficiency (ΦPSII) and non-photochemical quenching (NPQ) between the CK and the various drought treatment groups (PEG+PR, PEG+MR, PEG+DR). As the duration of the drought treatment increased, the Fv/Fm continued to decrease in all drought treatment groups, with significant differences between the groups (*p* < 0.05). Among them, the PEG+PR group showed the most pronounced decline in the Fv/Fm, with an accelerated decrease rate between 6 h and 8 h. In contrast, the decline trend of the Fv/Fm in the other two groups was relatively gentle. Over time, ΦPSII exhibited a general decreasing trend in all drought treatment groups. There were differences among different clones, with the PEG+DR group showing a relatively small decrease in ΦPSII throughout the entire drought treatment process. During the stress treatment, NPQ in all drought treatment groups initially increased and then decreased, with the trend of NPQ changes being generally similar across all treatment groups.

**Figure 6:**
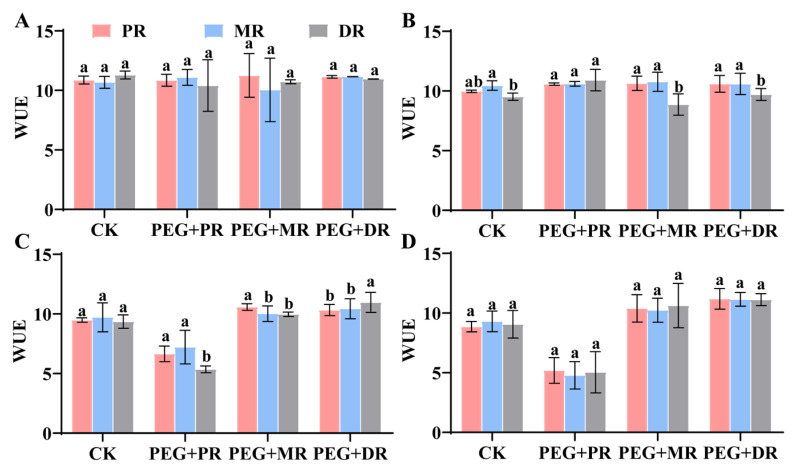
Changes in water use efficiency (WUE) of all clonal ramets under PEG6000 conditions over 2 h (**A**), 4 h (**B**), 6 h (**C**) and 8 h (**D**). CK stands for control group, PEG+PR means drought treatment of the PR of the clonal plant, PEG+MR means drought treatment of the MR of the clonal plant, and PEG+DR means drought treatment of the DR of the clonal plant. Data in the figures indicate mean of four replicates (±SD), and different letters on error bars indicate significant difference at *p* < 0.05.

In the 2 h drought treatment (Figure 6), there was a small difference in water use efficiency (WUE) between the CK and the drought treatment groups (PEG+PR, PEG+MR, PEG+DR). As the duration of drought treatment increased, the WUE of the other drought treatment groups, except for the PEG+PR group, showed an increasing trend, with significant differences between groups (*p* < 0.05). However, the WUE value of the PEG+PR group decreased after 6 h of the drought treatment.

### 2.5. Principal Component Analysis and Correlation Analysis of Gas Exchange Parameters

**Figure 7:**
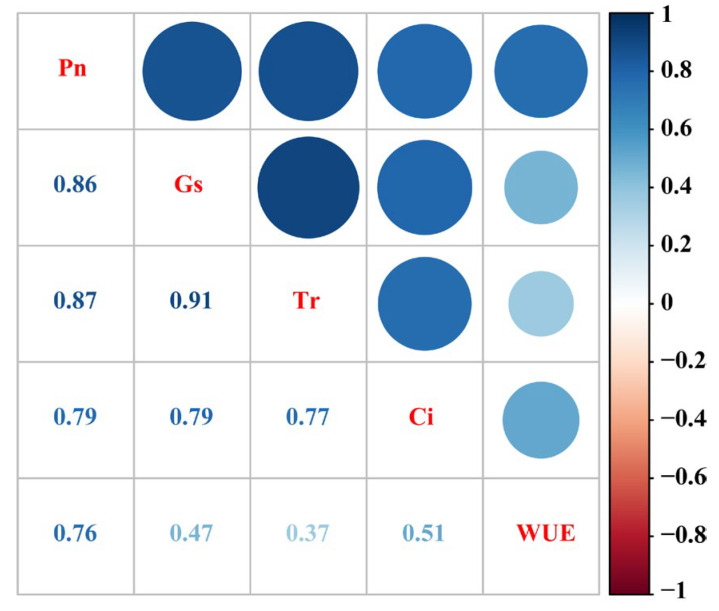
Correlation analysis of gas exchange parameters of clonal ramets.

This study revealed the complex relationships between the gas exchange parameters in Kentucky bluegrass clone ramets under drought stress. As shown in Figure 7, the Pn was significantly positively correlated with Gs and Tr (*r* > 0.85), indicating that stomatal behavior was a key factor regulating photosynthesis and transpiration. The strong positive correlation between Gs and Tr (*r* = 0.91) further supports this mechanism. Additionally, WUE showed a strong correlation with Pn but weaker correlations with Gs, Tr, and Ci (*r* = 0.37–0.51).

**Figure 8:**
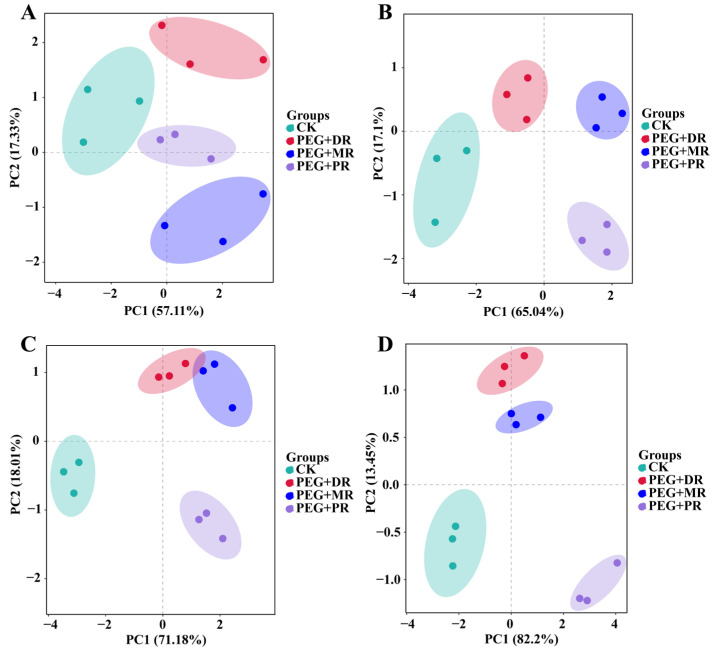
Principal component analysis (PCA) of physiological parameters of clonal ramets. (**A**) 2 h drought stress; (**B**) 4 h drought stress; (**C**) 6 h drought stress; (**D**) 8 h drought stress.

As drought stress duration increased, the dispersion among treatment groups changed (Figure 8). With prolonged drought exposure, the scores of PC1 gradually increased. The PEG+PR group consistently showed higher scores on PC1 across all time points, suggesting its photosynthetic capacity remained better maintained under drought stress. The PEG+DR group separated from others on PC2 but became closer to the that of PEG+MR group at 6 h and 8 h. The PEG+MR group occupied an intermediate position at 8 h, reflecting its drought adaptability between the PEG+PR and PEG+DR groups.

## 3. Discussion

The physiological integration of cloned plants is not a uniform occurrence, displaying considerable variation among species [18]. This suggests that both the presence of water sharing among the internodes of clonal plants and the patterns of such sharing in terms of space and time are specific to each species. Certain species, including *Fragaria orientalis* [19], *Thymus vulgaris* [20], and *Ipomoea aquatic* [21], exhibit significant physiological integration. Our study’s results suggest that Kentucky bluegrass is classified among those clones that demonstrate extensive physiological integration.

The stable isotope ^2^H tracer technique was used to analyze the water physiological integration of Kentucky bluegrass clonal ramets under drought stress conditions. In each ramet, the root system served as the entry point for ^2^H_2_O, and the δ^2^H value in the roots was significantly affected by the ^2^H treatment. However, the δ^2^H values in the various tissue parts of each ramet showed notable changes as the drought duration increased. This observation aligns with previous research indicating that drought can enhance a plant’s ability to absorb water [9,22]. In certain situations, physiological water integration occurs among Kentucky bluegrass clones, allowing water to be transported from the roots to the upper parts to alleviate water deficits during drought [16]. Initially, the roots of each ramet absorb ^2^H_2_O, which is then moved to the leaves and other tissues through exoplasmic or symplastic pathways [23,24].

The transfer of materials and energy between clones may result from their inherent source–sink relationships, in which the uneven distribution of resources and energy drives the integration of these clones. It is therefore hypothesized that the gradient of resources and energy between clones influences both the direction and intensity of their physiological integration [22]. This study found that the osmotic potential gradient among Kentucky bluegrass clones significantly impacts the direction and intensity of water physiological integration within the clonal system. Water transfer occurs from clones with high osmotic potential to those with low osmotic potential [19,25]. In our study, the ramets treated with PEG6000, which served as the introduction points of the ^2^H isotope, had the highest δ^2^H values. Moreover, whether the PR, MR or DR were treated with PEG not only affected the δ^2^H values of the stressed ramets themselves but also changed those of the other ramets. This indicates that there is a bidirectional water transfer trend among clonal ramets. While water transfer between the ramets of clonal plants can take place through physiological integration, the amount of water transferred from the source ramet to the stressed ramet is not unlimited [26]. The cloned ramets will only engage in limited water integration based on their own water needs and the level of water stress experienced by the ramet.

Water stress significantly affects many crucial physiological and ecological processes, particularly photosynthesis [13]. The findings indicate that water integration among clonal plants can notably enhance their photosynthetic performance and WUE, thereby supporting their survival and growth in suboptimal habitats [13]. The water integration in Kentucky bluegrass enhanced water availability and photosynthetic activity in clonal plants [18]. In this study, the Pn, Gs, and Tr all decreased with prolonged drought stress. Under drought stress, there were differences in the decline rates of Pn and Fv/Fm among different clonal ramets. The decline in the PEG+DR group was relatively slow, while that in the PEG+PR group was the most obvious. This might have been because the different positions of the clonal ramets led to differences in their responses and adaptation abilities to drought stress. When the PR ramet were subjected to drought stress, the photosynthetic system of the plants was more severely damaged, and the physiological integration ability was reduced. When the DR ramets were under drought stress, the PR and MR ramets might still have had relatively excellent water-maintaining abilities, which promoted physiological integration. After 2 h of drought treatment, there was little difference in WUE between the control and each drought treatment group. As the drought treatment time increased, the WUE of other drought treatment groups, except for the PEG+PR group, showed an upward trend. The WUE of the PEG+PR group decreased after 6 h of the drought treatment, which also indicated that the PR might have played a key role in the overall physiological integration of the plants. These results clearly demonstrate that the physiological integration of Kentucky bluegrass enhances the photosynthetic capabilities of clones under water stress, allowing them to sustain performance in challenging environments. Therefore, fostering physiological integration among clonal plants can facilitate their survival and growth in soils characterized by significant variability in water availability.

## 4. Conclusions

Our findings indicate that water physiological integration occurs among Kentucky bluegrass clones, exhibiting a trend of bidirectional water transfer. Additionally, this water integration helps to mitigate the negative effects of water scarcity on the photochemical activity of Kentucky bluegrass. The PEG+PR treatment group was most affected by drought stress, while the PEG+DR treatment group was least affected. In heterogeneous environments, clonal plants utilize physiological integration mechanisms to share resources among different ramets, thereby enhancing their adaptability to varying conditions, which holds significant ecological and evolutionary importance. Investigating the mechanisms of physiological integration and their regulation can provide a theoretical foundation for further exploring the potential applications of clonal plants.

## 5. Materials and Methods

### 5.1. Plant Material and Conditions

Kentucky bluegrass cultivar ‘Arcadia’ from United States Department of Agricultural was used for research. Two-year-old sod (tillering stage) of ‘Arcadia’ was collected from a horticultural experiment field of the Northeast Agricultural University (Harbin, Heilongjiang Province, China). The breeding process of the clone ramets was performed as previously described; in a clonal population with identical genotypes, plants with consistent growth conditions were selected, consisting of three clones. Based on the distance of each ramet from the mother ramet, they were designated as the proximal ramet (PR), middle ramet (MR), and distal ramet (DR) [23]. The plants were cultured under controlled recovery conditions (20 ± 5 °C, 12 h photoperiod/day, average light intensity of 1000 ± 20 μmol photons m^−2^s^−1^, relative humidity of 60 ± 5%) for 2 weeks in containers (length 16 cm × width 10 cm × height 20 cm), filled with river sand, and watered every three days to keep the plants in adequate moisture conditions. The ramets were given 50% full-strength Hoagland solution per week to ensure the basic nutrient supply of the plants.

### 5.2. Treatments

**Figure 9:**
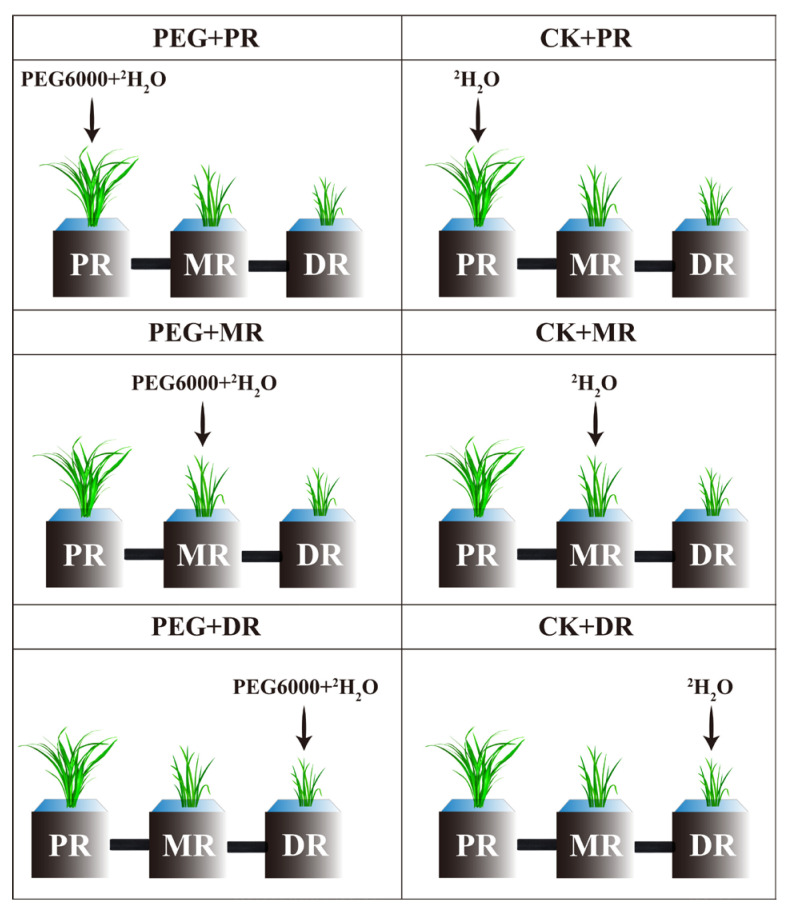
Experimental design diagram. The three drought stresses were PEG+PR (PEG6000 and ^2^H isotope added to PR ramet), PEG+MR (PEG6000 and ^2^H isotope added to MR ramet), and PEG+DR (PEG6000 and ^2^H isotope added to DR ramet). The corresponding CK only adds the ^2^H isotope. PR, MR and DR were connected by rhizome.

A conventional Hoagland solution was prepared according to previous studies [27], and ^2^H_2_O (produced by Shanghai Institute of Chemical Engineering) was used to substitute H_2_O to prepare the Hoagland solution with ^2^H isotope labeling; the osmotic potential of the solution was −0.03 MPa. Then, 200 g of PEG6000 was weighed and slowly added to the 1 L Hoagland nutrient solution labeled with the ^2^H isotope to adjust the osmotic potential to −1 MPa to simulate drought stress. After four weeks of recovery culture of ramets, plant materials with three clone ramets were selected, and the rhizomes were cleaned with distilled water, during which the rhizomes were not damaged. A slit was cut in the side of the pots (length 10 cm × width 10 cm × height 20 cm) and a hole was drilled at the bottom of the slit. The connected rhizomes were put through the holes, three ramets were separately planted, and gaps in the pots were sealed with glue to avoid loss of water. Then, the rhizomes with black polyethylene film were covered to prevent sunlight exposure. The Hoagland solution prepared with or without PEG6000 was poured into the designated pots, as shown in Figure 9. Different drought stress gradients (2 h, 4 h, 6 h and 8 h) were set up to stress the PR, the MR and the DR, respectively. The ramets not treated with PEG-6000 were the control group (CK). Each treatment group (CK, PEG+PR, PEG+MR, PEG+DR) was planted in three pots.

### 5.3. Analysis of ^2^H Isotope

The method of ^2^H determination is as described by predecessors [28]. The δ^2^H values in Kentucky bluegrass leaves and roots were determined with an isotope ratio mass spectrometer (Isoprime 100, Germany, Isoprime, Cheadle, UK) coupled to an elemental analyzer (vario PYRO cube, Elementar, Chester, UK) in continuous-flow mode at the Institute of Environment and Sustainable Development in Agricultural, Chinese Academy of Agricultural Sciences.

### 5.4. Determination of Leaf Gas Exchange Parameters

Leaf gas exchange parameter measurement was conducted using a portable photosynthesometer (Li 6400, Li-Cor Environmental, Bourne, MA, USA) between 9:00 am and 11:00 am when the gas exchange behavior was stabilized [29]. At 1000 μmol·m^−2^·s^−1^ light intensity, light adaptation for 10 min, determination of leaf gas exchange parameters was carried out, including net photosynthetic rate (Pn), transpiration rate (Tr), intercellular CO_2_ concentration (Ci) and stomatal conductivity (Gs). The Pn/Tr ratios obtained were the WUE of the plant [30,31].

### 5.5. Determination of Chlorophyll Fluorescence

Chlorophyll fluorescence was measured using IMAGING-PAM chlorophyll fluorometer (IMAGING-PAM, WALZ, Effeltrich, Germany) [32]. First, the leaves were subjected to dark adaptation for 30 min, followed by the measurement of initial fluorescence. After applying a saturating light pulse, the maximum photochemical efficiency (Fv/Fm) and maximum fluorescence were determined. Subsequently, the leaves were exposed to actinic light for 10 min, and the steady-state fluorescence and the maximum fluorescence under light adaptation were recorded, after which the non-photochemical quenching coefficient (NPQ) and effective quantum yield of photosystem II (ΦPSΙΙ) were determined.

### 5.6. Statistical Analysis

Data from each parameter are presented as mean ± SD. Analysis of variance was performed using SPSS (version 12.0 SPSS Inc., Chicago, IL, USA). The Shapiro–Wilk test in SPSS software was used to detect the normal distribution of the data. One-way ANOVA, and Student’s *t*-test were used to test the data at the 5% confidence level (*p* < 0.05). In addition, R software (version 4.4.3) was used for principal component analysis (PCA) and correlation analysis. GraphPad Prism (version 8.0 GraphPad Inc., La Jolla, CA, USA) was used to draw figures.

## Data Availability

Data are contained within the article.
